# Functional brain network organization measured with magnetoencephalography predicts cognitive decline in multiple sclerosis

**DOI:** 10.1177/1352458520977160

**Published:** 2020-12-09

**Authors:** Ilse M Nauta, Shanna D Kulik, Lucas C Breedt, Anand JC Eijlers, Eva MM Strijbis, Dirk Bertens, Prejaas Tewarie, Arjan Hillebrand, Cornelis J Stam, Bernard MJ Uitdehaag, Jeroen JG Geurts, Linda Douw, Brigit A de Jong, Menno M Schoonheim

**Affiliations:** Department of Neurology, Amsterdam UMC, Vrije Universiteit Amsterdam, MS Center Amsterdam, Amsterdam Neuroscience, Amsterdam, The Netherlands; Department of Anatomy & Neurosciences, Amsterdam UMC, Vrije Universiteit Amsterdam, MS Center Amsterdam, Amsterdam Neuroscience, Amsterdam, The Netherlands; Department of Anatomy & Neurosciences, Amsterdam UMC, Vrije Universiteit Amsterdam, MS Center Amsterdam, Amsterdam Neuroscience, Amsterdam, The Netherlands; Department of Anatomy & Neurosciences, Amsterdam UMC, Vrije Universiteit Amsterdam, MS Center Amsterdam, Amsterdam Neuroscience, Amsterdam, The Netherlands; Department of Neurology, Amsterdam UMC, Vrije Universiteit Amsterdam, MS Center Amsterdam, Amsterdam Neuroscience, Amsterdam, The Netherlands/Department of Clinical Neurophysiology and MEG Center, Amsterdam UMC, Vrije Universiteit Amsterdam, MS Center Amsterdam, Amsterdam Neuroscience, Amsterdam, The Netherlands; Donders Institute for Brain, Cognition and Behaviour, Radboud University, Nijmegen, The Netherlands; Klimmendaal Rehabilitation Center, Arnhem, The Netherlands; Department of Neurology, Amsterdam UMC, Vrije Universiteit Amsterdam, MS Center Amsterdam, Amsterdam Neuroscience, Amsterdam, The Netherlands; Department of Clinical Neurophysiology and MEG Center, Amsterdam UMC, Vrije Universiteit Amsterdam, MS Center Amsterdam, Amsterdam Neuroscience, Amsterdam, The Netherlands; Department of Neurology, Amsterdam UMC, Vrije Universiteit Amsterdam, MS Center Amsterdam, Amsterdam Neuroscience, Amsterdam, The Netherlands/Department of Clinical Neurophysiology and MEG Center, Amsterdam UMC, Vrije Universiteit Amsterdam, MS Center Amsterdam, Amsterdam Neuroscience, Amsterdam, The Netherlands; Department of Neurology, Amsterdam UMC, Vrije Universiteit Amsterdam, MS Center Amsterdam, Amsterdam Neuroscience, Amsterdam, The Netherlands; Department of Anatomy & Neurosciences, Amsterdam UMC, Vrije Universiteit Amsterdam, MS Center Amsterdam, Amsterdam Neuroscience, Amsterdam, The Netherlands; Department of Anatomy & Neurosciences, Amsterdam UMC, Vrije Universiteit Amsterdam, MS Center Amsterdam, Amsterdam Neuroscience, Amsterdam, The Netherlands; Department of Neurology, Amsterdam UMC, Vrije Universiteit Amsterdam, MS Center Amsterdam, Amsterdam Neuroscience, Amsterdam, The Netherlands; Department of Anatomy & Neurosciences, Amsterdam UMC, Vrije Universiteit Amsterdam, MS Center Amsterdam, Amsterdam Neuroscience, Amsterdam, The Netherlands

**Keywords:** Multiple sclerosis, cognitive functioning, magnetoencephalography, magnetic resonance imaging, longitudinal, network organization

## Abstract

**Background:**

Cognitive decline remains difficult to predict as structural brain damage cannot fully explain the extensive heterogeneity found between MS patients.

**Objective:**

To investigate whether functional brain network organization measured with magnetoencephalography (MEG) predicts cognitive decline in MS patients after 5 years and to explore its value beyond structural pathology.

**Methods:**

Resting-state MEG recordings, structural MRI, and neuropsychological assessments were analyzed of 146 MS patients, and 100 patients had a 5-year follow-up neuropsychological assessment. Network properties of the minimum spanning tree (i.e. backbone of the functional brain network) indicating network integration and overload were related to baseline and longitudinal cognition, correcting for structural damage.

**Results:**

A more integrated beta band network (i.e. smaller diameter) and a less integrated delta band network (i.e. lower leaf fraction) predicted cognitive decline after 5 years ($R_{\mathrm{adj}}^{2}=15\%$), independent of structural damage. Cross-sectional analyses showed that a less integrated network (e.g. lower tree hierarchy) related to worse cognition, independent of frequency band.

**Conclusions:**

The level of functional brain network integration was an independent predictive marker of cognitive decline, in addition to the severity of structural damage. This work thereby indicates the promise of MEG-derived network measures in predicting disease progression in MS.

## Introduction

Cognitive impairment (CI) occurs in 43%–70% of patients with MS, which profoundly affects their quality of life.^1^ An accurate prognosis of cognitive decline in MS is currently difficult, as the mechanisms underlying cognitive decline remain unclear. Structural brain damage, including gray matter (GM) atrophy and tissue integrity loss, predicts cognitive decline, but cannot fully explain the extensive heterogeneity found between MS patients.^2–4^

Recent cross-sectional studies have shown that disruptions in functional brain network organization may further elucidate mechanisms underlying CI in MS,^5^ even in the absence of atrophy.^6^ These studies have demonstrated that worse cognitive function could partly be understood in terms of changes in network integration (i.e. communication between spatially remote brain regions)^7–9^ and segregation (i.e. local connectedness).^10,11^

Functional network organization in MS has mainly been studied using functional (f)MRI, a technique based on metabolic changes. Another method to quantify functional networks is magnetoencephalography (MEG), which, as opposed to fMRI, directly measures neural activity.^12^ In addition, MEG has an excellent temporal resolution, and its spatial resolution has recently significantly improved.^12,13^ In fact, a recent MS study suggested that MEG has a higher sensitivity to detect cognitive relevant disruptions in functional networks than fMRI.^14^

Still, it remains unclear whether functional brain network disruptions can also predict cognitive changes over time in MS due to a critical lack of longitudinal studies. This study therefore investigated whether MEG-derived functional brain network measures can predict cognitive decline in MS patients over 5 years and whether these measures have an independent predictive value beyond structural brain pathology.

## Methods

### Participants

Data of 146 MS patients from the Amsterdam MS Cohort were included (67% women, age = 48.30 ± 11.16 years, disease duration = 12.95 ± 7.74 years; Table 1),^4^ and a subsample of this cross-sectional MEG data has been published before.^14^ Patients obtained MEG recordings, structural MRI, and a neuropsychological evaluation at baseline. In 100 of these patients, a neuropsychological follow-up assessment was acquired after 4.60 (±0.61) years. Disability at both time-points was classified using the Expanded Disability Status Scale (EDSS).^15^ Highest level of attained education ranged between 1 (did not finish primary school) and 7 (university degree) and was categorized into low (1–3), medium (4–5), and high (6–7). Sixty healthy controls from whom cognitive scores were obtained at baseline and follow-up (average time-interval 5.46 ± 1.08 years) were included to standardize cognitive scores for all participants (see details in the following section).^4^ Approval was obtained from the institutional ethics review board of the Amsterdam UMC (numbers 2004/9, 2012/140, and 2010/336), and participants gave written informed consent prior to participation.

**Table 1.** Demographic, clinical, cognitive, and MRI characteristics.

	Total patient group (*N* = 146)	Patient group with follow-up data (*N* = 100)	
	Baseline data	Baseline data	Follow-up data
Demographics			
Age; years, mean (SD)	48.30 (11.16)	48.40 (10.97)	53.00 (10.84)
Women; *n* (%)	98 (67.1)	69 (69.0)	69 (69.0)
Education; median (range)	4 (1–7)	4 (1–7)	4.5 (1–7)
Clinical characteristics			
MS type; RRMS/SPMS/PPMS (%)	76.6/12.4/11.0	80.0/13.0/7.0	69.0/23.0/7.0
Disease duration; years, mean (SD)	12.95 (7.74)	13.17 (7.48)	17.77 (7.32)
EDSS; median (range)	3 (0–8)	3 (0–8)	4 (0–8.5)
Cognitive scores, mean (SD)	*Z*-score	*Z*-score	Yearly rate of cognitive change
SRT—Verbal memory	−0.72 (1.14)	−0.77 (1.18)	−0.08 (0.24)
10/36 SPART-Visuospatial memory	−1.02 (1.26)	−1.01 (1.28)	−0.01 (0.27)
SDMT—Information processing speed	−1.46 (1.20)	−1.40 (1.18)	−0.04 (0.21)
MCT—Working memory	−1.33 (1.43)	−1.37 (1.48)	−0.03 (0.30)
WLGT—Verbal fluency	−0.81 (0.91)	−0.76 (0.86)	−0.04 (0.19)
CST—Executive function	−1.12 (1.42)	−0.99 (1.35)	−0.08 (0.27)
SCWT—Sustained attention and executive function	−0.94 (1.05)	−0.93 (1.10)	0.05 (0.25)
Average cognition	−1.07 (0.80)	−1.03 (0.80)	−0.03 (0.11)
MRI characteristics			
Deep gray matter volume; mL, mean (SD)	55.79 (6.39)	56.37 (6.27)	—
Cortical gray matter volume; L, mean (SD)	0.70 (0.05)	0.75 (0.06)	—
White matter lesion volume; mL, median (range)	10.04 (1.36–85.5)	8.56 (1.36–69.18)	—

SD: standard deviation; MS: multiple sclerosis; RRMS: relapsing remitting multiple sclerosis; SPMS: secondary progressive multiple sclerosis. PPMS: primary progressive multiple sclerosis; EDSS: Expanded Disability Status Scale; SRT: Selective Reminding Test; SPART: Spatial Recall Test; SDMT: Symbol Digit Modalities Test; MCT: Memory Comparison Test; WLGT: Word List Generation Test; CST: Concept Shifting Test; SCWT: Stroop Color-Word Test; MRI: magnetic resonance imaging.

Disease duration represents the disease duration since symptom onset.

### Neuropsychological evaluation

The neuropsychological assessment consisted of an extended version of Rao’s Brief Repeatable Battery of Neuropsychological tests (BRB-N),^16^ as described previously:^4^ (1) the Selective Reminding Test (SRT) assessed verbal memory; (2) the 10/36 Spatial Recall Test assessed visuospatial memory; (3) the Symbol Digit Modalities Test assessed information processing speed; (4) the Memory Comparison Test assessed working memory; (5) the Word List Generation Test assessed verbal fluency; (6) the Concept Shifting Test (CST) assessed executive function, particularly concept shifting; and (7) the Stroop Color-Word Test assessed sustained attention and executive function, including inhibiting an automated response.

Raw scores were adjusted for age, sex, and education based on a normative sample of healthy controls, converted into test-specific *z*-scores based on the means and standard deviations (SDs) of healthy controls and averaged into one cognitive score at baseline.^4,17^ To analyze cognitive decline over time, the modified practice adjusted reliable change index (RCI) was applied to correct for learning effects, as described previously (see Supplementary Information).^4,18^ RCIs were divided by the patients’ time interval, and test-specific yearly RCIs were averaged across tests into a “yearly rate of cognitive decline” representing longitudinal cognition.^4^

### MRI scans, lesion load, and GM volumes

At baseline, participants were scanned on a 3-Tesla whole-body MRI (General Electric Signa HDxt), including FLAIR and 3D-T1 sequences as previously described.^4^ Automated lesion detection using k-nearest neighbor classification was run on 3D-FLAIR images. Deep GM volumes were estimated using FIRST (FSL5) after lesion filling (using LEAP). Cortical GM volumes were calculated by masking deep GM areas from total GM segmentations from SIENAX (also FSL5). All GM volumes were multiplied with the so-called V-scaling factor, which describes the difference in skull size of each participant compared to the skull of the standard brain, using FSL.

### MEG recordings and pre-processing

**Figure 1.**
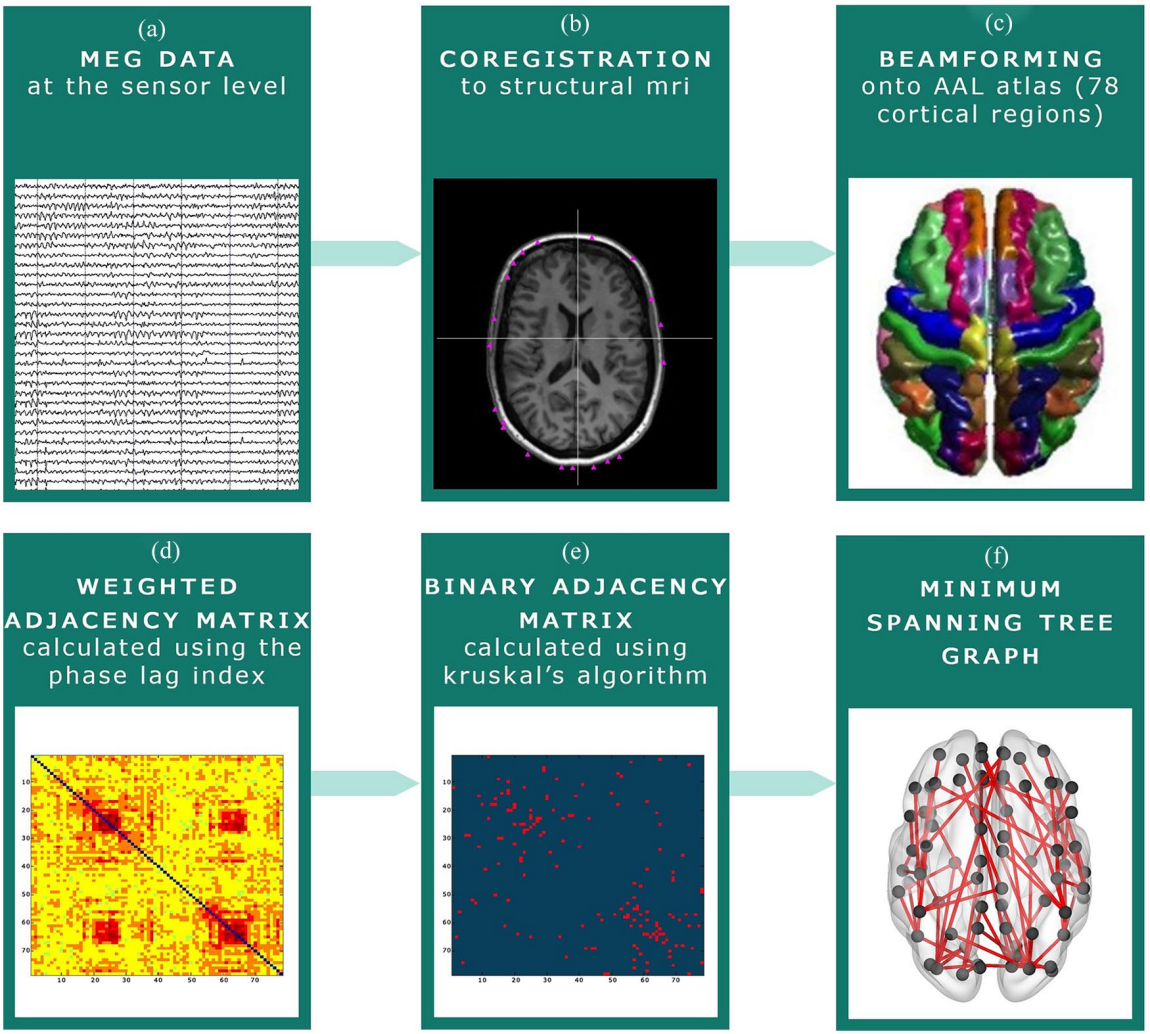
MEG pre-processing steps. (a) MEG recording at sensor level. (b) The MEG recording was co-registered to the participants’ structural MRI. (c) Beamforming was applied to convert the MEG signal to source space: signals were projected onto the Automated Anatomical Labeling (AAL) atlas. (d) The phase lag index (PLI) was calculated between each of the 78 cortical regions of the AAL atlas. (e) The Minimum Spanning Tree (MST) was constructed based on the PLI, which consists of the 78 strongest connections. These connections were subsequently binarized. (f) An example of an MST graph.

Five minutes of eyes-closed resting-state MEG data were recorded on a 306-channel whole-head system (Elekta Neuromag Oy, Helsinki, Finland) and processed according to a standardized procedure (Figure 1 and Supplementary Information). In short, MEG data were visually inspected to discard malfunctioning channels, and the temporal extension of Signal Space Separation removed artifacts.^19^ Source-localized MEG data were then constructed for 78 cortical regions of the automated anatomical labeling atlas^20^ using a beamformer approach.^13^ Subsequently, 52 epochs of 4096 samples (3.27 s) were filtered into canonical frequency bands in Matlab (R2012a): delta (0.5–4 Hz), theta (4–8 Hz), alpha1 (8–10 Hz), alpha2 (10–13 Hz), beta (13–30 Hz), and gamma (30–48 Hz).

### Functional connectivity and the MST

**Figure 2.**
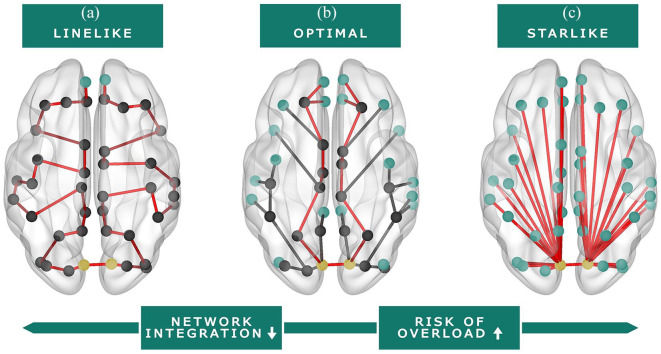
Visual representation of functional brain network organization (MST). Red line: representation of the diameter (i.e. longest shortest path of the MST). Green nodes: leaf nodes (i.e. a node with one connection). Yellow nodes: nodes with the highest betweenness centrality (i.e. the node with the largest fraction of shortest paths in the MST passing through that node). (a) Line-like network organization; this organization is considered inefficient and less integrated. (b) Balance between a line-like and star-like network; this organization is considered optimal. (c) Star-like network organization; this organization is considered efficient, but there is a larger risk of hub overload.

Functional connectivity between all 78 cortical regions was calculated with the phase lag index,^21^ which served as input for the minimum spanning tree (MST) algorithm (Supplementary Information provides more details).^22–24^ This resulted in a dichotomized backbone of the functional brain network formed by 78 cortical regions and only the 77 strongest functional connections, as the MST contains a fixed number of regions (i.e. nodes) and connections (i.e. edges).^22,24^ Consequently, there are no arbitrary thresholds, which optimizes comparability between participants.^25^ MST measures representing global network integration and overload (i.e. leaf fraction (LF), betweenness centrality (BC), diameter, tree hierarchy; Table 2; Figure 2) were calculated for each of the six frequency bands in Matlab using previously described codes.^9^

**Table 2.** Description of MST measures.^9,23^

Measure	Definition	Network integration and overload
Leaf fraction	The fraction of nodes in the MST with a degree (i.e. number of connections) of one.	A higher leaf fraction indicates a more ‘star-like’ network organization, which indicates *more network integration* as well as a *larger chance of overload* of central regions (Figure 2). The leaf fraction represents the dependency of the network on central nodes.
Maximum betweenness centrality (BC)	BC of a node quantifies the fraction of shortest paths in the MST passing through that node. The maximum BC represents the node with the highest BC.	A higher maximum BC indicates a more ‘star-like’ network organization, which indicates *more network integration* as well as a *larger chance of overload* of central regions (Figure 2). The higher the BC, the more important a node is within the network, but also the larger the chance that this node will be overloaded.
Diameter	The largest distance between any two regions of the MST network, which is normalized for the total number of connections.	A larger diameter indicates a more line-like organization, which indicates *less network integration* and a *lower chance of overload* of central regions (Figure 2). The diameter represents the efficiency of the information transfer across the network.
Tree hierarchy	The tree hierarchy measures the trade-off between large scale integration in the MST (measured with the leaf fraction) and the overload of central nodes, also called hubs (measured with the maximum BC).	A higher tree hierarchy indicates a more star-like organization, which indicates *more network integration* as well as a *larger chance of overload* of central regions (Figure 2). The tree hierarchy represents the hierarchal structure of the MST.

MST: minimum spanning tree; BC: betweenness centrality.

### Statistical analyses

Statistical analyses were performed in SPSS 22 and bootstrapping analyses in R 3.6.2.

#### Correlational and regression analyses

Pearson’s correlations were calculated between MST and MRI measures. White matter lesion load was log-transformed. Pearson’s partial correlations were calculated between brain measures (i.e. both MST and MRI measures) and cognition (i.e. baseline and longitudinal cognition), correcting for age, education, sex, and, for longitudinal analyses, also baseline cognition. Correlations with MST measures were Bonferroni-corrected at *p* < 0.008 (i.e. *p* < 0.05 divided by six frequency bands) and other correlations were set at *p* < 0.05.

Then, backward stepwise linear regression analyses were performed to identify the most important predictors of baseline and longitudinal cognition. Initial models allowed one predictor for every 10 participants (including covariates), and the strength of the partial correlations between the MST measures and cognition was used to select MST predictors for these initial models. Tree hierarchy was excluded due to collinearity with LF and maximum BC, as it is defined as their ratio.

The initial cross-sectional MEG model (*N* = 146) included 10 MST variables with the strongest partial correlations with baseline cognition, along with four fixed covariates (age, education (two dummy variables) and sex). The initial longitudinal MEG model (*N* = 100) included five MST measures with the strongest partial correlation with longitudinal cognition and five fixed covariates (age, education (two dummy variables), sex, and baseline cognition). Backward stepwise selection was applied on these models based on a threshold of *p* < 0.10, and *p* < 0.05 was considered statistically significant. Next, to assess the independence of MST predictors beyond structural damage, both cross-sectional and longitudinal final MEG-models were combined with structural measures, on which backward selection was applied again, resulting in final MEG-MRI models. Finally, as a post hoc exploration, the final longitudinal MEG-model was repeated using individual cognitive tests as outcome measures to assess how specific the set of MST predictors was for different cognitive functions.

#### Bootstrapped validation of linear regression models

To investigate the robustness of the MST predictors in our regression models, 10,000 bootstrap samples were created non-parametrically (i.e. observations drawn with uniform probabilities and replacement from the total sample). On each bootstrap sample, we performed a linear regression analysis (“enter”) including only variables selected in the final cross-sectional and longitudinal MEG and MEG-MRI models. In addition, each bootstrap sample was used to perform a full backward selection procedure on the initial cross-sectional and longitudinal MEG models, based on the Akaike’s information criterion (AIC),^26^ that balance model fit with its complexity (i.e. related to the number of predictors in the model). The fitting used a ‘step’-function with default settings from the car-package.^27^ From the thus acquired 10,000 models, the selection frequency of each MEG predictor (criterion > 50%)^28^ was reported.

## Results

### Baseline and follow-up characteristics

Table 1 presents an overview of baseline and follow-up characteristics. The largest rate of yearly cognitive decline was found for tests that measured executive function and verbal memory (both cognitive decline scores = −0.08). The 100 patients included in the longitudinal analyses did not differ from the 46 patients from whom no follow-up cognition data were obtained with respect to demographics, baseline cognition, and disease duration (*p* > 0.05).

### Cross-sectional correlates of structural damage

Out of all 24 MST measures, 16 were significantly related to deep GM volume and 18 to lesion volume (*p* < 0.008; Table 3). More specific, a lower LF, larger diameter, and lower tree hierarchy, representing a less integrated network, related to lower deep GM volumes and higher lesion volumes (Figure 2(a)). Also, a lower gamma BC related to higher lesion volumes. MST measures were not significantly related to cortical GM volumes (Table 3) and disease duration (*p* > 0.008).

**Table 3.** Correlations between MST measures and both cognition and structural brain measures.

MST measure	Cognition (partial *r*)		Structural brain measures (*r*)		
	Cross-sectional	Longitudinal	Deep GM volume	Cortical GM volume	WM lesion volume
Leaf fraction					
Delta	0.20^	0.19	0.35*	0.12	−0.33*
Theta	0.18^	<–0.01	0.27*	0.12	−0.32*
Alpha1	0.24*	0.07	0.25*	0.05	−0.28*
Alpha2	0.20^	0.10	0.35*	0.17^	−0.37*
Beta	0.22^	0.02	0.34*	0.13	−0.32*
Gamma	0.19^	0.11	0.38*	0.19^	−0.36*
Betweenness centrality					
Delta	<0.01	0.07	0.01	−0.09	−0.05
Theta	<–0.01	−0.02	0.14	0.06	−0.17
Alpha1	0.06	−0.04	<–0.01	−0.05	<0.01
Alpha2	−0.08	<0.01	0.06	0.05	−0.14
Beta	0.05	−0.02	0.08	−0.04	−0.13
Gamma	−0.08	0.04	0.16	0.03	−0.29*
Diameter					
Delta	−0.22^	−0.22^	−0.36*	−0.16	0.35*
Theta	−0.03	0.08	−0.20^	−0.03	0.25*
Alpha1	−0.19^	0.04	−0.13	−0.06	0.15
Alpha2	−0.09	−0.14	−0.26*	−0.12	0.24*
Beta	−0.15	0.14	−0.28*	−0.12	0.24*
Gamma	−0.11	−0.02	−0.27*	−0.14	0.33*
Tree hierarchy					
Delta	0.20^	0.15	0.35*	0.16^	−0.31*
Theta	0.20^	<–0.01	0.23*	0.11	−0.28*
Alpha1	0.23*	0.10	0.27*	0.08	−0.31*
Alpha2	0.27*	0.11	0.37*	0.17^	−0.35*
Beta	0.23*	0.02	0.34*	0.16	−0.30*
Gamma	0.25*	0.10	0.35*	0.21^	−0.27*

MST: minimum spanning tree; GM: gray matter; WM: white matter.

^ *p* < 0.05. *p < 0.008 (i.e. significant after correction for multiple comparisons). The partial correlations between cognition and the MST measures were corrected for age, education, sex, and for longitudinal cognition, also baseline cognition.

### Cross-sectional correlates of cognitive function

Worse baseline cognition was related to a lower LF and tree hierarchy in multiple frequency bands (*p* < 0.008; Table 3), which represented a less integrated network (Figure 2(a)). Worse baseline cognition also related to lower deep (partial *r* = 0.52, *p* < 0.001) and cortical (partial *r* = 0.50, *p* < 0.001) GM volumes, and a higher lesion load (partial *r* = −0.36, *p* = 0.001).

The initial cross-sectional MEG model included the LF (all frequency bands) and diameter (delta, alpha1, beta, and gamma bands). The final MEG model after backwards stepwise selection showed that a lower alpha1 LF (i.e. less integrated network; β = 0.24, *p* = 0.004) was the best correlate of worse cognitive performance ($R_{\mathrm{adj}}^{2}=10\%$; Table 4). This MST measure remained an independent correlate of cognition (β = 0.15, *p* = 0.041) in the cross-sectional MEG-MRI model, together with cortical (β = 0.33, *p* = 0.007) and deep GM volumes (β = 0.29, *p* = 0.009; $R_{\mathrm{adj}}^{2}\;\mathrm{model}=34\%$; Table 4).

**Table 4.** Regression models to predict cognitive decline and cognition at baseline.

	Final models		Bootstrapped validation final models		
	*B*	*P*	*B*_mean_	95% CI_mean_	*P*_median*_
Cognition at baseline (MEG model; $R_{\mathrm{adj}}^{2}=10\%$)					
Leaf fraction alpha1	13.99	0.004*	13.82	4.36–22.84	0.004*
*Sex*	*0.30*	0.029*	*0.30*	*0.04–0.57*	0.027*
*Age*	*–0.01*	*0.184*	*–0.008*	*–0.019 to 0.004*	*0.185*
*Education middle vs. low*	*0.25*	*0.141*	*0.25*	*–0.11 to 0.60*	*0.145*
*Education high vs. low*	*0.37*	0.025*	*0.37*	*0.04 to 0.68*	0.025*
Cognition at baseline (MEG and MRI model; $R_{\mathrm{adj}}^{2}=34\%$)					
Leaf fraction alpha1	8.79	0.041*	8.56	−1.36 to 18.08	0.043*
Cortical GM volume (L)	4.83	0.007*	4.85	1.42–8.12	0.006*
Deep GM volume (ml)	0.04	0.009*	0.04	0.008–0.06	0.008*
*Sex*	*0.15*	*0.204*	*0.16*	−*0.07 to 0.37*	*0.183*
*Age*	*0.01*	*0.064*	*0.01*	−*0.002 to 0.02*	*0.062*
*Education middle vs. low*	*0.31*	0.037*	*0.30*	−*0.02 to 0.63*	0.038*
*Education high vs. low*	*0.37*	0.010*	*0.36*	*0.05 to 0.69*	0.010*
Longitudinal cognitive decline (MEG model; $R_{\mathrm{adj}}^{2}=15\%$)					
Leaf fraction delta band	3.37	0.001*	3.40	0.94 to 5.93	0.001*
Diameter beta band	0.07	0.003*	0.07	0.03 to 0.10	0.003*
*Cognition at baseline*	*–0.02*	*0.148*	*–0.02*	*–0.05 to 0.01*	*0.158*
*Sex*	*0.03*	*0.264*	*0.03*	*–0.03 to 0.08*	*0.237*
*Age*	*–0.003*	0.011*	*–0.002*	*–0.004 to 0.001*	0.009*
*Education middle vs. low*	*0.04*	*0.190*	*0.04*	*–0.01* to *0.09*	*0.194*
*Education high vs. low*	*0.06*	0.031*	*0.06*	*0.01–0.11*	0.030*
Longitudinal cognitive decline (MEG and MRI model; $R_{\mathrm{adj}}^{2}=21\%$)					
Leaf fraction delta	3.35	0.001*	3.41	1.13–5.74	0.001*
Diameter beta	0.07	0.003*	0.07	0.03–0.10	0.003*
Cortical GM volume (L)	0.70	0.006*	0.69	0.24–1.16	0.006*
*Cognition at baseline*	*–0.04*	0.009*	*–0.04*	*–0.07 to 0.007*	0.010*
*Sex*	*0.02*	*0.363*	*0.02*	*–0.03 to 0.07*	*0.291*
*Age*	*–0.001*	*0.641*	*–0.001*	*–0.003 to 0.002*	*0.454*
*Education middle vs. low*	*0.05*	*0.076*	*0.05*	*0.003 to 0.10*	*0.079*
*Education high vs. low*	*0.07*	0.010*	*0.07*	*0.02 to 0.12*	0.010*

CI: confidence interval; MEG: magnetoencephalography; MRI: magnetic resonance imaging; GM: gray matter.

Covariates are presented in italics.

* Due to a skewed distribution of the *p* values across the 10,000 bootstrap samples, the median *p* value is noted.

These predictors remained significant when the final MEG and MEG-MRI models were bootstrapped (median *p* value <0.05; Table 4). Bootstrapped backward selection of the initial MEG model showed that all MST predictors were selected in a minority of the bootstrap samples (<50%). The alpha1 LF had the highest selection frequency (47.5%), and the selection frequency of the other MST predictors ranged between 22.7% and 44.1%.

### Predictors of longitudinal cognitive decline

The yearly rate of cognitive decline and MST measures at baseline were not significantly correlated (*p* > 0.008; Table 3). Lower deep (partial *r* = 0.24, *p* = 0.018) and cortical (partial *r* = 0.28, *p* = 0.007) GM volumes at baseline did show correlations with the yearly rate of cognitive decline, lesion volumes did not (*p* > 0.05).

**Figure 3.**
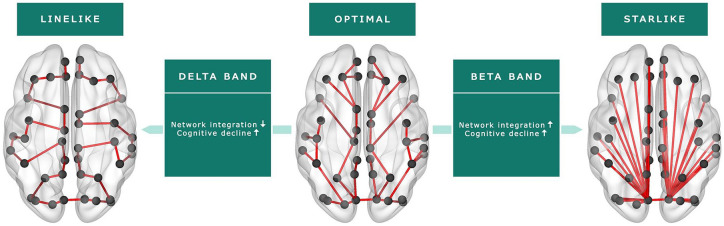
Functional brain network characteristics (MST) as predictors of cognitive decline. A less integrated delta band network (i.e. a more line-like organization) and a more integrated beta band network (i.e. a more star-like organization) at baseline predicted larger rates of yearly cognitive decline.

The initial longitudinal MEG model included the LF (delta and gamma bands) and diameter (delta, alpha2, and beta bands). The final MEG model after backwards selection showed that a lower delta LF (i.e. less integrated network; β = 0.40, *p* = 0.001) and a smaller beta diameter (i.e. more integrated network; β = 0.35, *p* = 0.003) predicted larger rates of cognitive decline ($R_{\mathrm{adj}}^{2}=15\%$; Table 4; Figure 3). These MST predictors remained independent predictors of cognitive decline (β = 0.39, *p* = 0.001 and β = 0.34, *p* = 0.003, respectively) in the longitudinal MEG-MRI model, together with lower cortical GM volume (β = 0.35, *p* = 0.006; $R_{\mathrm{adj}}^{2}\;\mathrm{model}=21\%$; Table 4).

These predictors remained significant when the final MEG and MEG-MRI models were bootstrapped (median *p* value <0.05; Table 4). Bootstrapped backward selection of the initial MEG model showed that the delta band LF and the beta band diameter were selected in 59.0% and 97.7% of the bootstrap samples, respectively, and the selection frequency of the other MST predictors ranged between 28.4% and 41.8%.

### Post hoc longitudinal analyses

The final longitudinal MEG model was repeated for separate cognitive tests and showed that the delta band LF only predicted cognitive decline on tests with the largest rate of decline (CST and SRT; *p* < 0.05). The beta band diameter did not reach significance (*p* > 0.05). The explained variance of all models was lower ($R_{\mathrm{adj}}^{2}=15\%$) than when a composite cognitive score was used.

## Discussion

This study aimed to investigate the predictive value of functional brain network characteristics on cognitive decline in MS patients. Our results showed that functional network integration and cortical GM volume at baseline predicted cognitive decline after a 5-year follow-up period. Importantly, functional network integration was an independent predictor of cognitive decline beyond structural brain pathology and therefore hold promise as a marker of imminent cognitive decline.

Our results indicate the value of MEG-based markers of network dysfunction in predicting cognitive decline in MS. Specifically, we found that both a less integrated delta band network, represented by a lower LF, and a more integrated beta band network, represented by a smaller diameter, predicted cognitive decline after 5 years. Both network changes may represent deviations away from the optimal network:^9,23^ a less integrated network indicates less efficient information transfer between spatially remote areas, while a more integrated network has a larger risk of overload of central brain regions, such as the thalamus and default-mode network.^24^ In line with this, network integration in a healthy population also shows frequency-specific differences: a previous study showed a high level of delta band integration and a low level of beta band integration in healthy controls,^29^ which is the reverse pattern that we found to predict cognitive decline in MS patients. It should be noted that functional network integration only predicted a modest amount of cognitive decline in our MS sample ($R_{\mathrm{adj}}^{2}=15\%$), which increased (to 21%) when also including cortical GM volume. Still, functional network integration was an independent predictor of cognitive decline in addition to cortical GM volume, indicating the added value of studying network functioning in the context of cognitive decline.

Our cross-sectional analyses showed that worse cognitive function was related to a less integrated network with fewer hub-like brain regions, as indicated by a lower tree hierarchy, which confirmed earlier MEG and fMRI studies.^7,9,10,23,30^ The direction and strength of this cross-sectional association was not specific to one frequency band, whereas our longitudinal results were highly frequency-specific. This could imply that network patterns heralding cognitive decline are different from those patterns identifiable after CI has already developed. This could indicate that the process of developing CI is related to a progressive spreading of network dysfunction across frequency bands over time. This finding that cross-sectional and longitudinal predictors of cognitive decline differ was also confirmed by a recent structural study, showing that while deep GM atrophy best relates to cross-sectional cognition, the primary predictor of future cognitive decline was actually cortical GM volume.^4^ This hypothesis also underlines the need for longitudinal MEG studies.

The underlying histopathological substrates of changes in functional network integration in MS patients remain unclear. In our study, functional network integration at baseline related to lesion load and deep GM volume, but not to cortical GM volume. Recent work suggested that functional network integration is likely to be facilitated by long-range white matter connections, including large commissural and association fibers, indicating that lesions within these long-range connections may lead to less integrated functional networks.^31,32^ Particularly these long-range connections seem to be vulnerable in MS,^32^ which may explain the widespread changes in functional network integration found in MS patients in our as well as other studies.^9,7^ Furthermore, as part of the deep GM, the thalamus seems to play a central role in functional network integration.^14,33^ This could possibly explain the observed relation between measures of network integration and specifically deep GM volume in our study. Future longitudinal studies need to elucidate whether damage in the long-range white matter connections, as well as thalamus atrophy, coincide with functional brain network changes or whether there is a certain order of events.

A limitation of our study is the relatively mild disease progression of our cohort, which could explain the modest amount of cognitive decline predicted by our functional and structural measures. Since the rate of decline differed between separate cognitive functions, we also investigated the predictive value of functional network integration for each cognitive function separately, but the explanatory power of our functional network predictors did not improve. In addition, we could not fully account for cognitive reserve, which may have a protective effect on cognitive decline.^2^ We did find that more highly educated patients had a lower rate of cognitive decline, but only including educational level may be too limited to represent cognitive reserve.^2,4^

Furthermore, a methodological consideration of network-based studies is the uncertainty of the computed network measures, given that several assumptions and choices need to be made.^34,35^ Still, an important advantage of MEG is that it directly measures the magnetic fields induced by neuronal currents and is therefore not affected by factors like neurovascular coupling, which strongly hamper interpretation of fMRI results.^12^ Moreover, we analyzed network integration based on the MST (i.e. the core of the functional brain network), which disregards weaker connections that are inherently more noisy. Although such weaker connections might still hold information, this algorithm avoids arbitrary choices with regard to thresholds or normalization procedures, which are usually needed when computing and comparing conventional network measures.^22–24^ We further validated our results with a bootstrap-based approach, which confirmed the robustness of our longitudinal results. A next step would be to validate our findings in different MS samples, as well as to employ multiple imaging modalities to study functional and structural brain network abnormalities in relation to cognitive decline.

To conclude, a combination of neurophysiological markers of network dysfunction and GM atrophy best predicted cognitive decline in MS. More specifically, our results indicate that both impaired functional network integration and lower cortical GM volume herald imminent cognitive decline, while white matter lesion load was not predictive. Interestingly, network dysfunction was not directly related to cortical atrophy, indicating the added value of including functional network measures when predicting decline in MS. As such, this work indicates the promise of network measures in predicting disease progression in MS patients, which warrants further study.

## Supplemental Material

sj-pdf-1-msj-10.1177_1352458520977160 – Supplemental material for Functional brain network organization measured with magnetoencephalography predicts cognitive decline in multiple sclerosis Click here for additional data file. Supplemental material, sj-pdf-1-msj-10.1177_1352458520977160 for Functional brain network organization measured with magnetoencephalography predicts cognitive decline in multiple sclerosis by Ilse M Nauta, Shanna D Kulik, Lucas C Breedt, Anand JC Eijlers, Eva MM Strijbis, Dirk Bertens, Prejaas Tewarie, Arjan Hillebrand, Cornelis J Stam, Bernard MJ Uitdehaag, Jeroen JG Geurts, Linda Douw, Brigit A de Jong and Menno M Schoonheim in Multiple Sclerosis Journal

sj-pdf-2-msj-10.1177_1352458520977160 – Supplemental material for Functional brain network organization measured with magnetoencephalography predicts cognitive decline in multiple sclerosis Click here for additional data file. Supplemental material, sj-pdf-2-msj-10.1177_1352458520977160 for Functional brain network organization measured with magnetoencephalography predicts cognitive decline in multiple sclerosis by Ilse M Nauta, Shanna D Kulik, Lucas C Breedt, Anand JC Eijlers, Eva MM Strijbis, Dirk Bertens, Prejaas Tewarie, Arjan Hillebrand, Cornelis J Stam, Bernard MJ Uitdehaag, Jeroen JG Geurts, Linda Douw, Brigit A de Jong and Menno M Schoonheim in Multiple Sclerosis Journal
