# Supplementary material for: Functional brain network organization measured with magnetoencephalography predicts cognitive decline in multiple sclerosis
Source: Mult Scler. 2020 Dec 9;27(11):1727–37. doi: 10.1177/1352458520977160 (PMC8474326; doi:10.1177/1352458520977160)
Supplement: sj-pdf-2-msj-10.1177_1352458520977160 – Supplemental material for Functional brain network organization measured with magnetoencephalography predicts cognitive decline in multiple sclerosis [file sj-pdf-2-msj-10.1177_1352458520977160.pdf]

SUPPLEMENTARY MATERIAL

METHODS

Cognitive decline over time

To analyze cognitive decline over time, the modified practice adjusted reliable change index (RCI) was applied to correct for learning effects, as described previously.[1, 2] The following equation was used: $((X_{i2} - X_{i1}) - (SEM_{HC2} - SEM_{HC1}))/S_{diff_{HC}}$, which represents the difference between the corrected participant scores on both two time-points (X_{i2}-X_{i1}), the difference between healthy controls' (HCs) standard error on both time-points (SEM_{HC2}-SEM_{HC1}), and the HCs' standard error of the difference between both time-points ($S_{diff_{HC}}$).[1, 2] The RCIs were divided by the participants' time interval and the test-specific yearly RCIs were averaged across tests into a 'yearly rate of cognitive decline' representing longitudinal cognition.[1]

MEG recordings and pre-processing

MEG data were acquired in a magnetically-shielded room using a 306-channel whole-head system (Elekta Neuromag Oy, Helsinki, Finland). Eyes-closed resting-state measurements were performed (5 minutes) at 1250 Hz, and two filters were applied online: 1) a 410 Hz antialiasing filter, and 2) a 0.1 Hz high pass filter. Signal Space Separation (xSSS) was applied to aid visual inspection (LB, SK and IN) of malfunctioning channels;[3] at most 12 channels were excluded for each participant. Then, the temporal extension of Signal Space Separation (tSSS) was performed offline in MaxFilter (Elekta Neuromag Oy, version 2.2.15) to remove artefacts.[4, 5] Figure 1 presents an overview of the MEG pre-processing steps.

The outline of the scalp was digitized with a 3D digitizer (Fastrak, Polhemus, Colchester, VT, USA) and head localization coils monitored the head position relative to the sensors, which were subsequently co-registered to the scalp surface obtained from MRI. The beamformer approach was applied to reconstruct source-localized MEG activity: each co-registered MRI was normalized to standard space after which an atlas-based beamformer approach (Elekta Neuromag Oy, version 2.1.28) was applied.[6] Cortical regions of the automated anatomical labeling atlas[7] were inversely transformed to each MRI, where centroids of these regions were selected to reconstruct localized MEG activity.[8] Subsequently, 52 consecutive epochs (based on the participant with the lowest number of epochs available) of 4096 samples (3.27 seconds), were included and band pass filtered in the canonical MEG frequency bands using a fast Fourier transform in Matlab (version R2012.a, Mathworks, Natick, MA, USA): delta (0.5-4 Hz), theta (4-8 Hz), alpha1 (8-10 Hz), alpha2 (10-13 Hz), beta (13-30 Hz), and gamma (30-48 Hz). All bins outside the pass band were set to 0, and accordingly, an inverse Fourier transform was used to obtain the filtered time series for the six frequency bands. The final number of included patients (N=146) was reached after the exclusion of eight additional patients, for whom MEG scans could not be included due to poor quality of the MEG data, which was the result of too many malfunctioning channels, failed head position monitoring, or regularly opening of the eyes. These eight participants were therefore not included in our sample.

Functional connectivity and the MST

Functional connectivity between all pair-wise combinations of the 78 cortical regions was assessed with the phase lag index (PLI),[9] also implemented in Matlab. The PLI is a measure of connectivity that quantifies the asymmetry of the distribution of phase differences ($\Delta\varphi$) between two time series, and ranges between 0 (no synchrony) to 1 (full synchronization).

Since the PLI only takes non-zero phase lag between two time series into account, the PLI is relatively insensitive to the effects of signal leakage.[9, 10] Connectivity matrices (78x78 per epoch for each participant) were fed to a minimum spanning tree (MST) algorithm, resulting in a dichotomized backbone of the functional brain network formed by only the strongest functional connections.[11-13] By definition, each MST contained 78 nodes (i.e. representing the 78 cortical regions) and a constant number of edges (77; N-1 for all MSTs),[11, 13] and therefore there is no need for arbitrary thresholds, which optimizes comparability between subjects.[14] Each MST was analyzed at baseline using the following measures that indicate aspects of network integration and the risk of network overload (see Table 2 for a more detailed explanation): leaf fraction (LF), betweenness centrality (BC), diameter, and tree hierarchy. These measures were calculated for each of the six frequency bands in Matlab using previously described codes.[15] A visual representation of these topological MST measures is represented in Figure 2.

REFERENCES

1. Eijlers AJC, van Geest Q, Dekker I, et al. Predicting cognitive decline in multiple sclerosis: a 5-year follow-up study. *Brain* 2018; 141: 2605-2618.

2. Iverson GL. Interpreting change on the WAIS-III/WMS-III in clinical samples. *Arch Clin Neuropsych* 2001; 16: 183-191. DOI: Doi 10.1016/S0887-6177(00)00060-3.

3. van Klink N, van Rosmalen F, Nenonen J, et al. Automatic detection and visualisation of MEG ripple oscillations in epilepsy. *Neuroimage Clin* 2017; 15: 689-701. DOI: 10.1016/j.nicl.2017.06.024.

4. Taulu S and Simola J. Spatiotemporal signal space separation method for rejecting nearby interference in MEG measurements. *Phys Med Biol* 2006; 51: 1759-1768. DOI: 10.1088/0031-9155/51/7/008.

5. Hillebrand A, Fazio P, de Munck JC, et al. Feasibility of clinical magnetoencephalography (MEG) functional mapping in the presence of dental artefacts. *Clin Neurophysiol* 2013; 124: 107-113. DOI: 10.1016/j.clinph.2012.06.013.

6. Hillebrand A, Barnes GR, Bosboom JL, et al. Frequency-dependent functional connectivity within resting-state networks: an atlas-based MEG beamformer solution. *Neuroimage* 2012; 59: 3909-3921. DOI: 10.1016/j.neuroimage.2011.11.005.

7. Tzourio-Mazoyer N, Landeau B, Papathanassiou D, et al. Automated anatomical labeling of activations in SPM using a macroscopic anatomical parcellation of the MNI MRI single-subject brain. *Neuroimage* 2002; 15: 273-289. DOI: 10.1006/nimg.2001.0978.

8. Hillebrand A, Tewarie P, van Dellen E, et al. Direction of information flow in large-scale resting-state networks is frequency-dependent. *Proc Natl Acad Sci U S A* 2016; 113: 3867-3872. DOI: 10.1073/pnas.1515657113.

9. Stam CJ, Nolte G and Daffertshofer A. Phase lag index: assessment of functional connectivity from multi channel EEG and MEG with diminished bias from common sources. *Hum Brain Mapp* 2007; 28: 1178-1193. DOI: 10.1002/hbm.20346.

10. Porz S, Kiel M and Lehnertz K. Can spurious indications for phase synchronization due to superimposed signals be avoided? *Chaos* 2014; 24: 033112. DOI: 10.1063/1.4890568.

11. Tewarie P, van Dellen E, Hillebrand A, et al. The minimum spanning tree: an unbiased method for brain network analysis. *Neuroimage* 2015; 104: 177-188. DOI: 10.1016/j.neuroimage.2014.10.015.

12. van Dellen E, Sommer IE, Bohlken MM, et al. Minimum spanning tree analysis of the human connectome. *Hum Brain Mapp* 2018; 39: 2455-2471. DOI: 10.1002/hbm.24014.

13. Stam CJ, Tewarie P, Van Dellen E, et al. The trees and the forest: Characterization of complex brain networks with minimum spanning trees. *Int J Psychophysiol* 2014; 92: 129-138. DOI: 10.1016/j.ijpsycho.2014.04.001.

14. van Wijk BC, Stam CJ and Daffertshofer A. Comparing brain networks of different size and connectivity density using graph theory. *PLoS One* 2010; 5: e13701. DOI: 10.1371/journal.pone.0013701.

15. Tewarie P, Hillebrand A, Schoonheim MM, et al. Functional brain network analysis using minimum spanning trees in Multiple Sclerosis: an MEG source-space study. *Neuroimage* 2014; 88: 308-318. DOI: 10.1016/j.neuroimage.2013.10.022.
